# *Cyclin D1* Gene Numerical Imbalances in Laryngeal Squamous Cell Carcinoma: A Tissue Microarray Grid Based Analysis

**DOI:** 10.31557/APJCP.2020.21.2.379

**Published:** 2020

**Authors:** Efthymios Kyrodimos, Vasileios Papanikolaou, Evangelos Tsiambas, Dimitrios Kikidis, Dimitrios Peschos, Vasileios Ragos, Nicholas Mastronikolis, Christos Riziotis, Aristeidis Chrysovergis

**Affiliations:** 1 *1st ENT Department, Hippocration Hospital, Medical School, University of Athens, *; 2 *Department of Pathology-Cytology, 401 GAH, *; 3 *Department of Pathology, 417 VA Hospital (NIMTS), *; 4 *Theoretical and Physical Chemistry Institute, Photonics for Nanoapplications Laboratory, National Hellenic Research Foundation, Athens, *; 5 *Department of Physiology, *; 6 *Department of Maxillofacial, Medical School, University of Ioannina, *; 7 *Department of Otorhinolaryngology, Head and Neck Surgery, Medical School, University of Patras, Greece. *

**Keywords:** Larynx, carcinoma, cyclin, protein, gene, grid

## Abstract

**Background:**

Deregulation of critical proteins involved in cell cycle stability, such as cyclins, is a frequent genetic event in the development and progression of solid malignancies. Concerning laryngeal squamous cell carcinoma (LSCC), *cyclin D1* oncogenic transformation leads to an aberrant protein expression and seems to affect the biological behaviour of the neoplasm. The aim of this study was to determine the correlation of *cyclin D1* numerical imbalances with the corresponding protein expression levels in LSCCs.

**Material and Method:**

Using tissue microarray (TMA) technology, fifty (n=50) histologically confirmed primary LSSCs were cored at a diameter of 1.5 mm. Immunohistochemistry (IHC) and chromogenic in situ hybridization (CISH) analyses were applied. Concerning the screening process in CISH slides, a novel real-time reference and calibration grid platform was implemented.

**Results:**

Protein overexpression was observed in 22/50 (44%) cases; whereas, gene amplification was seen in 13/50 (26%) cases (p=0.02). Combined protein/ gene deregulation was associated with the stage of malignancy (p= 0.0014, p=0.001), whereas overall protein expression was strongly correlated with the grade of tumour (p= 0.001).

**Conclusion:**

*Cyclin D1* gene amplification led to aberrant protein expression in LSCCs and it was also correlated with an aggressive biological behaviour. To best of our knowledge, this study was the first described grid based CISH analysis under conventional bright field microscopy for detecting gene numerical imbalances while providing a novel and accurate description for screening-mapping process in the entire slide area.

## Introduction

Laryngeal squamous cell carcinoma (LSCC) is the most common malignancy in larynx anatomical region. Main pathogenic factors associated with LSCC are tobacco and alcohol chronic consumption. Additionally, persistent viral (Human Papilloma Virus -HPV) infection is responsible for malignant transformation of the corresponding laryngeal epithelia (Mangano, et al., 2015). Gross Chromosome Instability (CI- polysomy/aneuploidy) and specific gene alterations (amplification, deletion, point mutations or epigenetic: aberrant promoter methylation) are critical genomic imbalances implicated in the development and progression of LSCC, as observed in solid malignancies with different origins (Grade et al., 2015; Polyak et al., 2009). In fact, these genetic events are observed even in hyperplastic and dysplastic laryngeal epithelia as early as genetic events. Among the genes involved crucially in the carcinogenetic process, cyclins represent important markers due to their influence on the cell cycle progression and stability (Bergshoeff et al., 2014). Activation of cyclins directly stimulates the progression of cell cycle and indirectly enhances cell proliferation via interactions with specific catalytic cyclin-dependent kinases (cdks). Members of *cyclin D* sub-family induce cell cycle progression by inducing retinoblastoma (Rb) protein phosphorylation. Especially, *cyclin D1 (CCND1* gene location: 11q13) acts at the middle of G1 phase by activating cdk4 (Almadori et al., 2002). *Cyclin D1 *overexpression is detected frequently in a variety of epithelial malignancies (Aboushousha et al., 2018; Tsiambas et al., 2007; Sawair et at., 2016). In the current retrospective experimental study, we co-analyzed *cyclin D1* numerical imbalances and the corresponding protein expression patterns in LSCC tissues. To best of our knowledge, this was the first study that described grid-based CISH analysis under conventional bright field microscopy for detecting gene numerical imbalances while providing a novel and accurate description for screening-mapping process in the entire slide area.

## Materials and Methods

*Patients and tissue specimens*

To perform this study, we obtained fifty (n = 50) formalinfixed and paraffin-embedded tissue samples of histologically confirmed primary LSCCs from patients who had undergone laryngectomy or hemilaryngectomy (including gross core biopsies in non- surgically resected cases) between 2007 and 2011 at the departments of Otorhinolaryngology- Head and Neck Surgery of the ‘‘Hippokrateion’’ and 417 Veterans Administration Hospital (NIMTS), Athens, Greece. All the patients were used to smoking. There were forty-five male patients with a median age of 61.5 years old and five female patients with a median age of 66.3 years old. For the control group, five specimens with histologically benign epithelia under conventional microscopy were also used. Given that no patients had a familial history of LSSC or inheritable cancer syndromes, the carcinomas were characterized as sporadic. Additionally, none of them had a history or positive DNA analysis for HPV infection. The local ethical committee consented to the use of these tissues (within the pathology department of the 417 Veterans Administration Hospital) for research purposes according to World Medical Association Declaration of Helsinki guidelines (2008). The tissue samples were fixed in 10 per cent neutral-buffered formalin. Haematoxylin and eosin (H and E) stained slides of the corresponding samples were reviewed to confirm histopathological diagnosis. All lesions were graded and staged according to the histological classification criteria presented by World Health Organization (WHO) and TNM staging system for head and neck cancers (Barnes et al., 2005). Clinicopathological data of the samples are demonstrated in Table 1.

*Tissue microarray (TMA) construction*

Areas of interest were identified in H&E stained slides by a conventional microscope (Olympus BX-50, Melville, NY, USA). Selection of those areas was performed on the basis of tumour sufficiency while avoiding sites of necrosis or bleeding. Having used ATA-100 apparatus (Chemicon International, Temecula, CA, USA), all of the source blocks were 1.5-mm diameter tissue cylindrical cores were transferred from each conventional donor block to the recipient blocks. After 3-mm microtome sectioning and H and E staining, the final constructed TMA blocks contained cores of tissue cylindrical specimens regarding the examined lesions carcinomas and benign epithelia. We observed microscopically that all of the examined samples were represented by tissue spots, confirming the adequacy of the examined specimens.

*Antibody and IHC assay*

**Figure 1:**
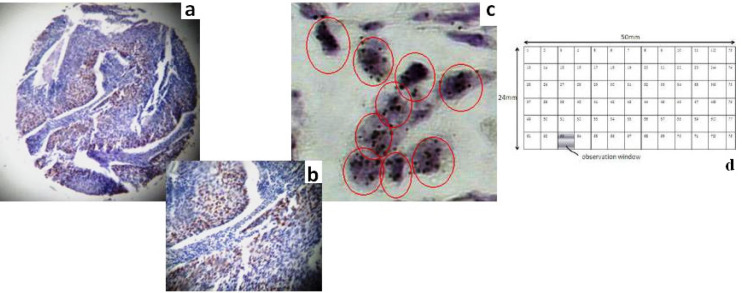
Cyclin D1 IHC and CISH Analyses on TMA Cylindrical Cores a. A LSCC tissue core demonstrates protein overexpression. Note a significant number of dark brown stained nuclei (IHC SCORE 3, anti-cyclin D1, DAB chromogen; original magnification40x) b. The same core is shown at magnification of 100x c. A LSCC tissue core demonstrates gene amplification. Note multiple scattered or in clusters gene signals (inside red circles DAB chromogen; original magnification 400x) d. Schematic presentation of the grid-based cover slip that was implemented in CISH slide screening-mapping. Note this prototype grid consisting of seventy- two (n=72) rectangular square areas arranged in 12 columns and 6 rows

We selected and applied a specific mouse monoclonal antibody anti-cyclin D1 (clone DSC6- Dako, Glostrup, Denmark) at dilution of 1:50. IHC protocol was carried out on 3-lm-thick paraffin section of the corresponding block. Tissue microarray sections initially deparaffinized in xylene, rehydrated via graded ethanol, and microwave-treated in target retrieval solution, pH 9.9 (Dako, Glostrup, Denmark). Immunostaining process for the applied marker was based on EnVision (DAKO, Glostrup, Denmark) assay using an automated staining system (I 6000-Biogenex, CA, USA) and according to corresponding manufacturer’s instructions. This specific assay is based on a soluble, dextran-polymer system, preventing endogenous biotin reaction and increasing the quality of stained slides. Briefly, the sections, after peroxidise blocking, were incubated with primary antibody for 30–60 min, depending on the corresponding antibody, at room temperature and then incubated with Horseradish peroxidase labeled polymer-HRP LP for 30 min. The antigen–antibody reaction was visualized using 3, 3’-diaminobenzidine tetrahydrochloride (DAB) as a chromogen substrate. Finally, tissue sections were slightly counterstained with hematoxylin for 30s, dehydrated, and mounted. For negative control slides, the primary antibody was omitted. Nuclear and perinuclear- cytoplasmic expression was acceptable for cyclin D1 protein. Staining intensity levels (score 0-3) were evaluated by two independent observers (pathologists). Conflicting observations were low (< 6%) for all made evaluations. Score 2 and 3 were considered positive for cyclin D1 overexpression (Figure 1a-b).

*Probe and CISH assay*

*Cyclin D1* gene status was determined using the ready to use SPOT LIGHT CYCLIN D1 DNA Probe (Zymed/InVitrogen, San Fransisco, USA). This digoxygenin-labeled probe is located on 11q13 and covers the entire gene area. Two slides were rinsed in deionised water and then placed in a coplin jar containing CISH FFPE Pretreatment Buffer (CISH Tissue Pre-treatment Kit, Zymed/InVitrogen, San Fransisco, USA). For heat pretreatment, the coplin jar was capped, loosely screwed, placed in a pressure cooker, and timed for 10 min after the pressure built up. The slides, then, were immediately washed in deionised water followed by enzyme digestion, which was performed by covering the sections with pepsin (CISH Tissue Pre-treatment Kit, Zymed) for 5 min at 37^o^C. The slides were washed with deionised water, dehydrated with graded ethanol, and air-dried. Ready to use digoxigenin-labeled *Cyclin D1 *gene was applied. Twenty microliters of probe was rinsed to each of the TMA sections. The tissue sections containing the added probe were denatured by placing the slides in a polymerase chain reaction (PCR) machine equipped with a slide block at 94^o^C for 5 min. The slides were then placed in a moist slide box and incubated at 37^o^C for overnight hybridization. The sections were stringently washed in 0.59 standard saline citrate at 75^o^C for 5 min. The CISH Polymer and the Horseradish (HRP) Detection Kit (Zymed/InVitrogen, San Fransisco, USA)-containing similar steps to IHC-were used. Shortly afterwards TMA sections were placed in 3% H_2_O_2_ and diluted with methanol for 10 min to block endogenous peroxidase. To block unspecific staining, Cas BlockTM (Zymed/InVitrogen, San Fransisco, USA) was applied and incubated for 10 min. Following incubation with mouse anti-dig for 30 min and then polymerised HRP conjugated anti-mouse for 30 min, the probe was visualized by DAB development (CISH Polymer Detection Kit, Zymed). TMA sections were lightly counterstained with hematoxylin and dehydrated in graded ethanol. At the end of the process, CISH gene copies were easily visualized as dark brown scattered or in small clusters dots, using a conventional, bright-field microscope (Figure 1b). Interpretation of *Cyclin D1* gene signal results was based on Zymed’s Evaluation Chart for CISH. According to this guide, two gene copies per nucleus demonstrated normal gene pattern; whereas, 6–10 or small clusters characterized a low-level gene amplification. High gene amplification level was characterized by the presence of more than 10 gene copies or clusters of them per nucleus in more than 50% of the examined cells (Figure 1c). In borderline cases, a second slide should be analyzed for chromosome 13 centromere copies in order to determine pure gene amplification or polysomy. In the current CISH TMAs CISH analysis, borderline cases were not identified.

*Slide screening process*

Screening procedure regarding the CISH stained slides was derived under bright field microscopy (microscope Olympus CX-31, Menvile, NY, USA, with ToupView image analysis software, ProWay/ToupTek Protonics, Cn/USA) with combined 100X/400X magnification. Concerning the CISH slides, screening was based on a set of cover slips integrated with spatial rectangular grid (now GCS) in order to perform a systematic way of slide eye-scanning. We used in this study a novel technique of micromachining with the use of ultra short Laser pulses at the femtosecond pulse duration regime (Femtosecond Laser Micromachining-FLM) in combination with high precision translation systems (Tsiambas and Riziotis, 2017). Laser techniques can allow direct writing and transfer of predetermined patterns by means of surface or sub-surface micromachining in a variety of materials ranging from glasses to soft polymeric materials. The technique of FLM inscription was applied here for the first time towards the fabrication of a visible rectangular grid in a microscope CISH slide’s cover slip for medical diagnostic purposes.

Adjusting the laser writing characteristics the inscripted grid lines’ width could be typically ranging from 5μm to 500μm. We used commercially available cover slips with length of 50mm, width of 24mm, and thickness 0.5mm (Menarini, IT), which were made by typical borosilicate based glass. This prototype grid consists of seventy- two (n=72) rectangular square areas arranged in 12 columns and 6 rows. Each square segment has a typical surface area of 4mm x 4mm equal to 16mm^2^. Each square segment can have appropriate indexing marks printed with various techniques like laser based micromachining in a suitable way to assure minimum visual interference under microscope inspection. In the present study, the indexing was provided by sequential numbering of the square cells as indicated schematically in Figure 1d. In the specific example due to the selection of grid’s cells size, appear also at the right part of the grid six residual non-rectangular cells, numbered at the figure as 73-78 that do not affect of course the generalization of the proposed grid’s architecture. The grid’s size and cells’ density could be modified according to screener’s needs but here it was selected a specific size believing to be a well balanced choice according to our experience. At the end of the technical grids’ fabrication process, a new set of five (n=5) GCS was constructed. 

*Statistical analysis*

Descriptive statistics were performed for data analysis. Associations between variables including protein expression levels, gene copies numerical variants, and clinicopathological parameters such as sex, tumor grade and stage, anatomic location, and alcohol consumption were studied using Pearson Chi square test (χ^2^) estimated along with its 99% CI (SPSS v20 (SPSS Inc, Chicago, IL, USA). Two-tailed p-values ≤0.05 were considered statistically significant. Results and correlations (p-values) are described in Table 1. 

**Table 1:** Clinicopathological Parameters and Total Cyclin D1 IHC & CISH Results

Clinicopathological parameters		IHC		P-value	CISH		P-value
	(n=50)	OE	LE		Amplified	Non Amplified	
LSCC		22/50 (44%)	28/50 (56%)		13/50 (26%)	37/50 (74%)	
Gender				0.201			0.234
Male	45 (90%)	21/50	24/50		12/50	33/50	
Female	5(10%)	1/50	4/50		1/50	4/50	
Anatomical location				0.44			0.92
Supraglotis	5 (10%)	2/50	3/50		1/50	4/50	
Transglotis	45(64%)	20/50	25/50		12/50	33/50	
Grade				0.001			0.74
1	4(8%)	2/50	2/50		0/50	4/50	
2	19 (38%)	10/50	9/50		5/50	14/50	
3	27 (54%)	10/50	17/50		8/50	19/50	
Stage				0.0014			0.001
I	0(0%)	0/50	0/50		0/50	0/50	
II	4(8%)	2/50	2/50		0/50	4/50	
III	25(50%)	5/50	20/50		1/50	24/50	
IV	21(42%)	15/50	6/50		12/50	9/50	
Alcohol consumption				0.22			0.009
Yes	43(84%)	19/50	24/50		10/50	33/50	
No	7(16%)	3/50	4/50		3/50	4/50	

LSCC, Laryngeal Squamous Cell Carcinoma; IHC, Immunohistochemistry; OE, Over expression; LE, Low expression; CISH, Chromogenic in situ hybridization.

## Results

All of the examined cases were evaluated properly according to IHC analysis, demonstrating different cyclin D1 expression levels. Increased (Score 2 and 3) protein expression was observed in 22/50 (44%) tissue cores regarding LSCCs. Overall *cyclin D1* expression was associated to stage and grade of the examined patients (p=0.001, p=0.0014, respectively); whereas, no other statistical correlations were identified concerning the rest clinicopathological parameters (gender: p=0.201, anatomic location: p=0.44, alcohol consumption p=0.22). Concerning CISH grid-based screening analysis, 13/50 (26%) tissue cores demonstrated low to high gene amplification (isolated multiple gene copies >=6 and gene clusters), while the rest 37/50 (64%) were found to be normal diploid (2-3 signals per nucleus). Overall gene numerical imbalances (amplification) were associated strongly with the stage of the tumours and the history of alcohol consumption (p=0.001, p=0.0092); whereas, no other statistical correlations were identified concerning the rest clinicopathological parameters (gender: p=0.234, anatomic location: p=0.92, grade: p=0.74). Comparing IHC and CISH results, gene amplification was correlated to protein overexpression (p=0.02). 

## Discussion

Extensive histogenetic analyses have shown that CI combined or not with specific oncogene over activation and suppressor gene inactivation is correlated with the progression of histopathologically confirmed premalignant lesions (dysplasia) to LSCC in laryngeal epithelia (Hanken et al., 2014). In fact, CI is strongly correlated with an increased risk for malignant transformation in hyperplastic and moderate to severe dysplastic epithelia. Focusing on *cyclin D1 *(*CCND1* gene band) amplification, based on Fluoresence in situ hybridization (FISH) and Array GeneChip analyses several studies observed that significant proportions of protein overexpressed cases demonstrate this molecular specific mechanism of gene deregulation. Interestingly, the highest rates of cyclin D1 gene amplification are observed in Head and Neck Squamous Cell Carcinoma (HNSCC), especially regarding not LSCC, but also pharyngeal and Oral Squamous Cell Carcinomas (OSCCs) (Niméus et al., 2014).

In the current study, we applied a combined IHC and CISH protocol in order to explore the role of protein and gene *cyclin D1* deregulation in LSCC TMAs. Although CISH molecular method is a fine alternative to FISH for analyzing tissues or TMAs in solid malignancies, there are very limited data on LSCC, in contrast to other malignancies such as breast carcinoma (Sui et al., 2014; Wang et al., 2014; Lundgren et al., 2012). Our research study showed that simultaneous protein and gene deregulation was associated with the stage of malignancy; whereas, overall protein expression was strongly correlated with the grade of tumour. Additionally, gene amplification seems to be a significant genetic mechanism leading to protein overexpression in these malignancies. Concerning the current molecular results, another study observed that although a significant proportion of examined LSCCs demonstrated cyclin D1 gene amplification, the main anatomic location in HNSCCs characterized by this mechanism was the hypo-pharyngeal epithelia, an indirect evidence of a site-specific prevalence for oncogene amplifications (Blessmann et al., 2013). Furthermore, another study co-analyzed CCND1 numerical imbalances and a specific A/G polymorphism in exon 4 based on differential and RFLP- Polymerase chain reaction (PCR) assays, respectively. They reported D1 A870G polymorphism and increased susceptibility for LSCC development at the glottic region (Verim et al., 2013). Similarly, another study reported carriers of cyclin D1 G870A and G1722C polymorphisms, especially among smokers, were characterized by increased risk for developing HNSCC (Lin et al., 2014), which is another paradigm of a site-specific prevalence regarding cyclin D1 oncogene transformation. Based on these studies, it seems that gene amplification, although significant,- is not the only cyclin D1 deregulation mechanism. Finally, the current experimental study was characterized with the pilot implementation of an innovative reference and calibration grid on conventional cover slips in the screening process of CISH slides. We already reported this improved technique as a tool for systematic screening in immunocytochemistry stained slides (Tsiambas et al., 2018). 

In conclusion, our combined IHC and CISH analysis on LSCC TMAs showed that cyclin D1 gene amplification was a strong deregulation mechanism leading to aberrant over-expression of the corresponding protein product. In addition, it was correlated with aggressive phenotype in a subset of them. Controversial published results regarding *cyclin D1* gene expression patterns in LSCC should be explained at the basis of selecting patients with different clinico-histogenetic characteristics. In conjunction to this, grid-based CISH analysis, under conventional bright field microscopy, is an easy to use molecular method for specific detecting genetic signatures in LSCC patients based on gene numerical imbalances.
